# Mixed methods research on reflective writing in teacher education

**DOI:** 10.3389/fpsyg.2024.1394641

**Published:** 2024-10-09

**Authors:** Michaela Gläser-Zikuda, Chengming Zhang, Florian Hofmann, Lea Plößl, Lisa Pösse, Michaela Artmann

**Affiliations:** ^1^Institute for Educational Science, University of Erlangen-Nürnberg, Erlangen, Germany; ^2^Institute for Children and School Education, University of Gießen, Gießen, Germany; ^3^Department of School Research, University of Cologne, Cologne, Germany

**Keywords:** mixed methods, qualitative content analysis, computational linguistics, reflective writing, teacher education

## Abstract

In this paper, mixed methods research is presented and discussed in the context of research on reflective writing for the professional development of pre-service teachers. First, we present prominent theoretical frameworks to analyze reflective writings. Second, we review relevant methodological approaches of research on reflective writings in teacher education, such as qualitative, quantitative and mixed methods. Third, we present a study from our research lab combining qualitative content analysis and linguistic analyses as an example for a concurrent mixed method approach. The results of the qualitative content analysis indicated that 198 reflective writings of pre-service teachers were primarily descriptive and on a low level. Computational linguistic analyses revealed that affective and cognitive terminology utilization in reflective writing differed significantly across the different levels of reflection, with a higher frequency of such terms correlating with deeper levels of reflection. Thus, essential challenges and opportunities of implementing such a mixed method study to analyze reflective writings are illustrated and discussed. Finally, we conclude the paper by discussing on how mixed methods approaches might be further advanced in the field of reflective writing research in teacher education.

## 1 Introduction

Reflective practice is widely recognized as a pivotal educational strategy for professional development, especially within higher education (Chan and Lee, 2021; Ryan, 2013). It plays a crucial role in several aspects, such as enhancing the understanding of professional knowledge (Korthagen, 2001), developing metacognitive skills (Desautel, 2009), and increasing self-awareness (Van Beveren et al., 2018). Specifically, within the domain of teacher education, reflection acts as a vital bridge between theoretical knowledge and practical application (Korthagen, 2018).

In research on reflective writing in teacher education, predominantly both quantitative and qualitative approaches are applied (Alsina et al., 2019; Cengiz et al., 2014; Houston, 2016; Lee and Abdul Rabu, 2022; Mena-Marcos et al., 2013). Quantitative methodologies are remarkably esteemed for their replicability, as they implement standardized measurements, enabling the consistent validation of research findings across diverse contexts, thereby bolstering the reliability of these outcomes. However, the inherent data simplification within quantitative methods might lead to omitting crucial nuances and in-depth information. Qualitative content analysis complements the limitations of quantitative methods. Unlike quantitative approaches that simplify data through numerical conversion, qualitative content analysis leverages detailed narratives to construct a nuanced understanding of educational phenomena. Consequently, the depth of insight qualitative research provides into educational practices and the comprehension of complex pedagogical phenomena offers invaluable perspectives for enhancing teacher education and the learning process. Furthermore, technological advancements offer possibilities for an automated analysis of reflective writing (Chong et al., 2020; Gibson et al., 2016). Automated analytics, encompassing Natural Language Processing (NLP) (Cui et al., 2019; Liu et al., 2020) and Machine Learning (ML) (Fan et al., 2017; Kovanović et al., 2018; Ullmann, 2019), provide a proficient and efficacious means to process and analyze large volumes of textual data. These technologies facilitate the identification of patterns, affective tendencies, conceptual relationships, and the depth and breadth of reflection within texts, thus significantly empowering educational researchers with unprecedented analytical capabilities. Consequently, combining automated analytic techniques with traditional quantitative and qualitative research methodologies represents a mixed methods approach.

This paper presents a mixed-method study from our research lab to bridge the gap in current methodologies for analyzing reflective writings in teacher education. The primary objective of this research was to enhance the depth and breadth of understanding of pre-service teachers’ reflective writing by employing qualitative content analysis and computational linguistics techniques. Specifically, we addressed the following research questions:

RQ1: How do qualitative content analysis and computational linguistics methods contribute to understanding reflective writings?

RQ2: In what ways can the integration of findings from these two approaches provide a more comprehensive insight into the quality of reflective writing?

We collected pre-service teachers’ reflective writings and applied qualitative content analysis to identify reflection levels. Subsequently, computational linguistics techniques were utilized to assess the complexity and structure of the texts quantitatively. By integrating the insights gained from both methods, our study offers a novel perspective that overcomes the limitations of using a single methodological approach. This dual-analysis framework provides a deeper understanding of the reflective writings.

## 2 Literature review

### 2.1 Definition of reflection and theoretical framework

When implementing an innovative and effective professionalization process for teachers, the reflective practitioner is increasingly cited as the target dimension (Argyris and Schön, 2008; Schön, 1983). Larrivee argues similarly, characterizing teachers as “social mediators, learning facilitators” and, above all, “reflective practitioners” (Larrivee, 2000, p. 293). In this light, reflective thinking is viewed as a relevant part of professional development, lifelong learning, and improving professional learning of (pre-service) teachers (Schön, 1983). However, decades after John Dewey’s (1933) important publication „*How We Think* “reflective thinking has become an umbrella term that encompasses a wide range of approaches and aims but lacking a clear definition, rationale, process, and outcome for pre-service teachers. According to Schön (1983), reflective thinking is the process of actively and critically thinking about one’s learning and experiences to understand and learn from them. It involves considering one’s thoughts and actions, and the context in which they took place to gain insights and make improvements. In addition to this, Boud et al. (1985, p. 19), Cleary et al. (2013, p. 69), and Gentile (2012, p. 102) have argued that reflection also has an affective component. The concept of reflection (concerning teacher’s professional development) has many different meanings and different approaches and directions in terms of its impact and benefits. Schön (1983) claims that education and reflective thinking practices are inseparable, and he introduces the notion of reflection-on-action (a social act) and reflection-in-action (a solitary act). Dewey (1933) and Schön (1983, 1987) argue that (pre-service) teachers learn more deeply when they reflect on experience with practice, and that transformational learning does not necessarily happen without reflective thinking processes. Cochran-Smith and Lytle (2009) focus from a practical perspective on the teacher’s professional development role, arguing that teachers must engage in ongoing reflective practice to improve their teaching skills. This approach advocates for teachers to develop as professionals to improve their effectiveness as teachers constantly.

In teacher education programs, numerous attempts were made to encourage pre-service teachers to engage in reflective practice (Borko et al., 1997). In orientation to the frameworks mentioned above, various models of reflection in professional education were developed (Bengtsson, 1995; Calderhead, 1992; Hatton and Smith, 1995). These models aim to reduce the complexity of reflection by representing it, for example through rubrics (Miller-Kuhlmann et al., 2016) or coding systems (Poldner et al., 2014), and to assess reflection levels (Larrivee, 2008). The analytical models commonly used in assessing reflective writings emphasize the depth or breadth of reflection (Ullmann, 2019). For example, the model developed by Hatton and Smith (1995) targeted assessment of depth of reflection, notably in teacher education. The model categorizes reflection into four progressive stages, from basic descriptive writing to descriptive reflection with justifications, dialogic reflection exploring internal dialogs, and culminating in critical reflection that considers broader historical, societal, and political contexts. In addition, the concept of reflection has also been framed as a hierarchical model (e.g., Ip et al., 2012; Kember, 1999; Mezirow, 1991). These models extended from levels of non-reflection to stages characterized by profound and high reflection. Conversely, a more multifaceted and dynamic model of reflection is evident in breadth models (e.g., Jung and Wise, 2020). An example is Gibbs’s (1988) Reflective Cycle, which outlines a six-stage framework for examining reflective texts. This cycle begins with a “description” stage, briefly summarizing the event, followed by “feelings,” where emotional reactions are articulated. The “evaluation” stage involves assessing the positives and negatives of the response. This leads to “analysis,” where understanding and interpretation of the event occur. The cycle then moves to “conclusions,” drawing general or specific lessons, and concludes with an “action plan,” outlining future responses in similar situations. Additionally, the breadth model has been utilized in research by various scholars, including, for example, Kolb (1984), Mansvelder-Longayroux et al. (2007), and Poldner et al. (2014).

### 2.2 Benefits and challenges of reflection in teacher education

The systematic review study by Van Beveren et al. (2018) demonstrates the significant role of reflection in contributing to learners’ professional development. As Korthagen (2001) highlighted, reflection enables (pre-service) teachers to connect their practical experiences with theoretical teaching knowledge. To support professional development, various strategies, such as reflective writing have been used in teacher education to facilitate pre-service teachers’ reflection during their professionalization. Reflective writing in teacher education typically focuses on the one hand on professional knowledge, linking learned theory to practice, and on the other hand on personal development, reflecting on learning achievements and planning for future actions. However, engaging in reflection about one’s learning and the teaching profession can be particularly challenging for pre-service teachers, and studies illustrate that the level of reflection among pre-service teachers is relatively low (Fütterer et al., 2024; Zhang et al., 2023). For example, reflections from less experienced teachers tend to be superficial and predominantly descriptive (Nguyen et al., 2014). Körkkö et al. (2016) noted that student reflections were more detailed after related practice but often lacked critical reflective thinking. Therefore, fostering reflection, especially in pre-service teachers, presents a challenge (Saric and Steh, 2017).

Furthermore, evaluation and research methods for reflective writing are problematic in various ways (Boud, 2010; Sumsion and Fleet, 1996). Assignments to levels of reflection or dimensions of reflection depend, among other aspects, on the research question, the sample or the existing data material, and the research methods applied. Consequently, evaluating reflections is not an absolute statement, mainly if only a specific research method is used (Kember et al., 2008). In order to analyze reflections from different perspectives and in both their depth and width and as accurately as possible, a combination of different methods is required (Whyss, 2013). Finally, when assessing the reflective skills of (pre-service) teachers, the research trend is clearly toward mixed methods approaches (Waag, 2017).

### 2.3 Methodological approaches of research on reflective writings

As described, the analysis of reflective writings is a highly challenging task. Qualitative and quantitative methods are applied in research in higher education and teacher education. In terms of qualitative methods, case studies are a strategy of inquiry in which the researcher explores in depth an event, activity, or process of one or more individuals. Well-known analysis methods in qualitative research are the documentary method (Bohnsack, 2013; Payne and Payne, 2004) and grounded theory (Glaser and Strauss, 1999). For example, based on a grounded theory coding system, Jumpakate et al. (2021) developed a typology of reflective writings to investigate novice teachers’ reflective acts to support their professional development. But to analyze reflective writings, predominantly qualitative content analysis is used (Krippendorff, 2004; Mayring, 2022; Mayring, 2000; *cf.* Gläser-Zikuda et al., 2020). Qualitative content analysis offers several coding strategies that are more or less theory-guided for a deductive or inductive coding process (Mayring, 2022). The object of qualitative content analysis can be any recorded or written communication, i.e., transcripts of interviews, protocols of observation, and written documents, in general. Not only the manifest content of the material is analyzed, but also the so-called latent content by an interpretative procedure, as well as formal aspects of the material, such as length, structure, etc. (Mayring, 2000). Three distinct analytical strategies (summary, structuring, and explication) may be carried out independently or in combination, depending on the research question (Mayring, 2000).

For the analysis of reflective writings, numerous studies applied qualitative content analysis. For instance, Dunne (2019) used qualitative content analysis and focused on analyzing the depth of reflection in their studies on reflective writings, providing valuable insights into the complexity of reflective thinking. Alt et al. (2022) and Bowman (2021) made substantial contributions by exploring the elements that make up reflective writing, shedding light on its constituent parts. Moreover, Pryjmachuk et al. (2019) identified emergent topics within reflective writings by using a qualitative content analysis. For example, Van Leeuwen et al. (2009) studied students’ personal experiences, emotions, and cognitive transformations by qualitatively examining their reflective writings. This method focuses intently on the nuanced interpretation of textual data, uncovering internal motivations, emotional variances, and cognitive transitions within the students’ reflective journeys. The potential of qualitative content analysis in research on reflective writings is related to identifying topics of reflection, the description of situations and experiences, individuals’ thoughts and feelings related to reflection, and the description and interpretation of the broadness and depth of reflection.

Not only qualitative methods but also quantitative methods are used in research on reflective thinking and practice. For instance, Poldner et al. (2014) devised a quantitative analysis framework based on existing literature and applied this framework to conduct quantitative content analysis. Additionally, they utilized statistical techniques to investigate various characteristics and patterns within reflective writing. This approach did not merely facilitate the comparison of reflection levels among individuals or groups but also delved into potential correlations between the depth of reflection and other pedagogical variables, such as teaching effectiveness (Azimi et al., 2019). Quantitative methods are generally applied for testing objective theories by examining the relationship among variables using statistical procedures, for example, based on questionnaires, e.g., Reflective Practice Questionnaire (RPQ) (Priddis and Rogers, 2018), Reflective Teaching Inventory (RTI) (Akbari et al., 2010) or Teacher’s Reflective Thinking Questionnaire (RTTO) (Choy and San Oo, 2012). The RPQ, represents an instrument promoting reflection processes, provides the results of a reflection, and, through the processing and the results, stimulates further and deeper reflections (Priddis and Rogers, 2018). In sum, an instrument was designed that can be applied for promoting reflection processes in different subjects and domains without an adaptation. Results from previous studies have shown that the implementation of the RPQ supports reflective practice and fosters confidence and further a desire for self-improvement (Priddis and Rogers, 2018). The Reflective Teaching Inventory (Akbari et al., 2010) and the Teacher’s Reflective Thinking Questionnaire (Choy and San Oo, 2012) were designed differently: Both questionnaires were specifically developed as a teacher reflection instrument and, in addition to cognitive, also for metacognitive, affective, critical and moral dimensions of reflection. In the RTI, several items are specifically aiming at didactic topics, as the instrument primarily focuses on teaching English. In contrast, the RTTQ may be used for different subjects and grades, as well as at different types of schools. The RTTQ has been proven to be a reliable and valid instrument for the assessment of reflective thoughts and it can contribute to improve students’ reflections (Choy and San Oo, 2012).

Quantitative methods, such as the mentioned questionnaires, are basically related to statistical analyses with many variables, including complex analyses that allow causal interpretation of data. This type of research provides a quantitative description of opinions or actions related to reflection and reflective writings of a sample with the intent of generalizing from a sample to a population (Creswell, 2015). But there is a lack of research on reflective writing in teacher education utilizing mixed methods research, as mentioned for example by Hayden and Chiu (2015). Therefore, in order to take the potential of both qualitative and quantitative methods for the analysis of reflective writings into account, we argue to combine qualitative content analysis with quantitative methods, which offer possibilities to investigate especially linguistic features playing an essential role in the analysis of reflective writings (Ullmann, 2019). Natural Language Processing and Machine Learning allow the analysis of a large amount of linguistic data and, beyond, a rapid analysis. For NLP, the Linguistic Inquiry and Word Count (LIWC) tool is a popular choice for methodology in research on reflective writing, as shown in studies by Cui et al. (2019), Savicki and Price (2015, 2021), and Springer and Yinger (2019). LIWC’s ability to analyze various linguistic attributes has made it valuable in these investigations.

A meanwhile well-known approach that systematically combines qualitative and quantitative methods is mixed methods research (Creswell, 2015; Tashakkori and Teddlie, 2010). Mixed methods research aims to leverage the strengths of both quantitative and qualitative methods to offer a more comprehensive understanding of complex phenomena, such as reflective writings in teacher education. In the last years, mixed methods research has been conducted in numerous studies in research in education and instruction, as well as teacher education (for an overview, e.g., Gläser-Zikuda et al., 2012; Hagenauer and Gläser-Zikuda, 2019; Mejeh et al., 2023).

### 2.4 Potential strengths of mixed methods research

Mixed methods research is more than simply collecting and analyzing both kinds of data; it also involves using both approaches together so that the overall strength of a study is more significant than just qualitative or quantitative research (Creswell and Plano-Clark, 2007). Different procedures for mixed methods inquiry strategies have been developed (Creswell and Plano-Clark, 2007). The following three main strategies are often applied. First, the sequential exploratory strategy involves a first phase of qualitative data collection and analysis, followed by a second phase of quantitative data collection and analysis that builds on the results of the first qualitative phase. The first qualitative phase is an exploratory step in a new research field to gain relevant aspects for a theoretical perspective to be developed. The purpose of this strategy is to use quantitative data and results to quantify the qualitative results and to support the interpretation of qualitative findings together with the quantitative results. Another example of this strategy is using qualitative findings to develop quantitative research instruments, such as questionnaires (Creswell and Plano-Clark, 2007).

Second, the sequential explanatory strategy goes in the opposite direction. It is a strategy for mixed methods design often applied by researchers with a strong quantitative focus. It is characterized by the collection and analysis of quantitative data in the first phase of research, followed by the collection and analysis of qualitative data in the second phase. This second phase aims to get deeper insight or interpret the, e.g., not expected quantitative results in more detail. Thus, the two data types are separate but connected concerning the research conclusions. This strategy often has a theoretical perspective, and in the first phase, quantitative methods are applied to test hypotheses.

The third strategy is the concurrent mixed methods approach; the researcher collects both quantitative and qualitative data concurrently, and then the two databases are compared to determine if there is convergence, differences, or at least some combination for interpretation of the data. This comparison may be seen as confirmation, disconfirmation, cross-validation, or corroboration (Greene et al., 1989). In this model, quantitative and qualitative methods are used to address the weaknesses inherent within one method with the strengths of the other. In this model, the quantitative and qualitative data collection are concurrent, meaning they are done in the same research study phase. The qualitative and quantitative parts and steps are equal. The mixing in this model is usually applied in the interpretation and discussion section in merging the data or integrating or comparing the results of two databases side by side in a discussion, for example, with quantitative statistical results and qualitative case studies that support or disconfirm the quantitative results (Creswell and Plano-Clark, 2007).

In this study, we employed mixed methods research to analyze the reflective writing of pre-service teachers. Our choice was primarily based on three factors: the complexity of reflective writing, the complementarity of qualitative and quantitative methods, and the state of the art in the research field. Firstly, reflective writing is a complex and multi-layered process involving pre-service teachers’ self-examination and critical thinking about their learning and teaching practices (e.g., Körkkö et al., 2016). This type of writing goes beyond merely narrating learning and teaching experiences; it delves deeply into reflections on beliefs about learning and teaching, instructional methods, and outcomes. Secondly, using mixed methods gave us a more comprehensive understanding and explanation of the research phenomenon in this situation. In this study, through qualitative content analysis, we detailed and categorized the reflective writings of pre-service teachers. This analysis helped us to construct a preliminary framework of reflective writing, which served as a foundation for subsequent quantitative analysis. We then used the LIWC2015 software to analyze these writings focusing on linguistic characteristics quantitatively. This step enabled us to statistically analyze a large volume of text data, revealing patterns in reflective writing. Finally, existing research indicates that mixed methods research are widely applied in education research. However, there is a lack of research on reflective writing in teacher education utilizing mixed methods research.

## 3 Method and results

### 3.1 Participants and data corpus

To analyze pre-service teachers’ reflective writing, we applied the concurrent mixed methods approach. In our study, we examined reflective writings of pre-service teachers in different teacher education programs (primary school, lower and higher secondary school) at one German university (Zhang et al., 2023). The reflective writings focused on core teacher education topics, namely pedagogical diagnostics (one week) and classroom management (three weeks). In total, 100 pre-service teachers participated in the study. Regarding the demographic composition of the sample, a significant majority, 71.90%, were female students, and 87.83% of the participants were in their first to third semesters (*M* = 1.71; *SD* = 1.68) of their respective teacher education programs. The students received a task with a case study for each topic and were asked to reflect upon these and to write one text for each topic.

**Figure 1:**
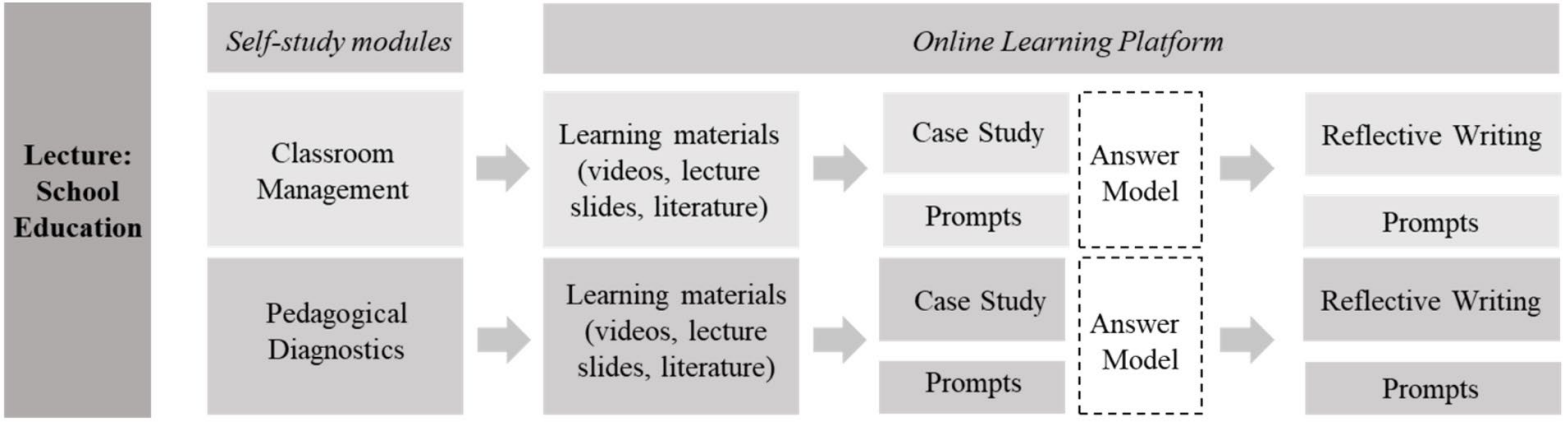
Mini-portfolio design and implementation (Zhang et al., 2023, p. 30).

Figure 1 shows the mini-portfolio applications.

For reflective writing assignments, students were required to reflect first on the professional content they had learned and second on their individual learning process. To guide and support students’ reflection process, structured prompts were designed. The prompts provided students with timely and focused information. The design of the prompts was based on Narciss’ (2006) framework, starting from the lowest level, KTC (knowledge of task constraints), to KH (knowledge of how to proceed). The prompts were primarily cognitive, and they were also very concrete because prompts with too abstract representations are difficult to achieve any effect (Berthold et al., 2009; Table 1).

**Table 1:** Illustrates examples of the prompts (for the topic classroom management) designed for this study.

**Prompt 1** KTC – Knowledge on task constraints Notes on subtasks and task requirements	**“I need a little food for thought…”** The topic of classroom management and dealing with disruptions can be divided into two large sub-areas: After clarifying what constitutes a teaching disruption (definition), it can be structured as to what happens before (preventive measures) and what happens after (intervention measures) of a possible disruption. The first part of the question (How do you analyze the following situation?) refers to prevention measures and situation analysis, the second part of the question (How do you react?) to intervention measures.
**Prompt 2** KC – Knowledge about concepts Notes on technical terms and context of terms, explanations of terms	**“I need a little tip regarding content…”** For example, relevant definitions and classifications for the terms “teaching disorders” and “conflicts” come from Becker (2006). To prevent conflicts, different actions can be taken; according to Osher et al. (2010) can be differentiated into three different categories. A well-known preventive approach comes from Kounin (1970), for example. On the intervention side, Becker’s (2006) action matrix is often used for conflict analysis and resolution.
**Prompt 3** KH – Knowledge on how to proceed Task-specific solution tips	**“I need more concrete advice…”** On the StudOn learning platform The folder for topic area 4 “Class management and dealing with disruptions” is located next to the slides for the lecture. A video recording of the lecture on the topic can also be viewed under the “Useful information and materials” tab (path: “Lecture recordings” ⋄ “Videos on topic area 4: Class management and dealing with disruptions”). Further information can be found in the “Literature” folder. The following book excerpts are particularly recommended: Becker (2006): Action matrix for conflict analysis and teachers solve conflicts. Lohmann (2011): Getting along with students. Ophardt and Thiel (2013): Classroom management.

They were offered to the students via the learning platform of the university.

A random sample of 200 reflective writings of 100 pre-service teachers (each student had to write a reflective text for two topics) was initially selected for analysis. However, after excluding an outlier (One students did not write reflective writing.), the final dataset comprised 198 reflective texts. Our analysis revealed that the average word count of these 198 texts was 230 words (*M* = 229.85; *SD* = 148.99). The number of words differed because there were no specifications regarding the length: The most extended reflection consisted of 806, the shortest of just 30 words.

### 3.2 Reflective level classification using qualitative content analysis

In a first step, reflective writings were analyzed using qualitative content analysis (Gläser-Zikuda et al., 2020). More specifically, we carried out a structured content analysis. The theoretical framework established by Hatton and Smith (1995) and adapted by Fütterer (2019) served as the theoretical background. Hatton and Smith’s framework for levels of reflection provides a systematic approach to categorizing and assessing the various levels of teacher reflection. This framework allows to accurately identifying and describing the specific characteristics and levels within (pre-service) teachers’ reflective writing. Many studies have used this model to evaluate reflective practices in (pre-service) teachers’ professional development, confirming its effectiveness and applicability. The coding system, and the deductive analysis procedure including coding rules and anchor examples are described in more detail by Zhang et al. (2023).

Each text was coded focusing on sentences and sections presenting relevant information about reflection based on the coding system presented. Depending on the overall coding result (in terms of representation of reflection level based on most of the codings in the different parts of the text), we rated each text at the end one of the four levels of reflection. Coders were members of the project team and were trained prior to formal coding. The intercoder reliability was checked by two coders who analyzed 198 reflective writings independently. The summative agreement between the coders was measured using Cohen’s Kappa, and the results indicate a very high level of agreement, with a Cohen’s Kappa score of 0.97 for pedagogical diagnostics and 0.96 for classroom management. Agreement was achieved when both researchers rated the whole the text on the same level of reflection. Disagreement occurred only in a few cases due to ratings on different levels of reflection. The codings of sentences and sections itself were not part of the intercoder reliability check. For further details of coding agreement and disagreement respectively, we described an example shown in the attachement. The theoretically derived category system used in our study is shown in Table 2. See Table A1 in the Appendix.

**Table 2:** Coding system of levels of reflection based on a structured content analysis (translated from the original German coding system applied in the study).

Category	Theoretical description	Anchor examples (from reflective writings)
Level 0: Descriptive writing	A situation (action, behavior…) is described. No efforts to classify or explain exist. Because reflective processes were defined as metacognitive, a mere description does not represent a reflective process.	“I have addressed the attributes of educational diagnostics and ways of classifying diagnostic instruments, but not the aspects of ‘types of performance assessment,’ ‘evaluation research,’ and the different forms of performance measurement.” (Topic: Pedagogical diagnostics)
Level 1: Descriptive reflection	Situations are either justified (personal judgment, perspective), or feelings, optional perspectives, or influential variables are reported, but without connecting them or considering their contextual embedding. Personal assumptions are presented.	“I found some diagnostic approaches, but should have created a better future perspective for the students and the teacher. For this purpose, specific examples, such as an observation matrix or a reference to the learning development diagnostic tools would have been good.” (Topic: Pedagogical diagnostics)
Level 2: Dialogic reflection	Different perspectives, influencing factors, and justifications for situations are identified. Perspectives are weighed in an interpersonal dialog. For this to happen, subjective theories and beliefs must become conscious. Competing perspectives are weighed up, leading to judgment.	“The scheme represents a well-structured approach to dealing with conflicts in everyday school life, but it seems to be designed mainly for conflicts among students. A model specifically for class – teacher conflicts might be more suitable for the task.” (Topics: Classroom management)
Level 3: Critical reflection	It is recognized that both situations and the identified perspectives, influential factors, and rationales are embedded in and influenced by a broader context (including historical, social, and political). Values and norms of the profession’s goals are also challenged, and institutional expectations are included.	“In addition, mathematics is a particular case here; the diagnostics must be different than in my subjects of study English, Geography, and Social Studies.” (Topics: Classroom management)

The main results of the qualitative content analysis of pre-service teachers’ reflective writings show that most of the reflective writings were on the descriptive level. Specifically, 37 reflective writings were categorized at the descriptive level, while a more significant number of 129 reflective writings were found to be at the descriptive reflection level, as detailed in Table 3. Among the 198 analyzed reflective writings of pre-service teachers, only 31 reflective writings were identified as engaging in dialogic reflection, indicating a more interactive and in-depth level of reflection. Notably, just one reflective writing text was coded at the highest level of reflection (critical reflection), which involves deep analytical and critical thinking.

**Table 3:** Distribution of pre-service teachers’ reflective writings to the reflection levels by learning topics.

	Description	Descriptive reflection	Dialogic reflection	Critical reflection
Pedagogical diagnostics	20	61	18	1
Classroom management	17	68	13	0
Total	37	129	31	1

### 3.3 Psycholinguistic features in reflective writings

To extract psycholinguistic features in the reflective writings, we employed the LIWC2015 method, a dictionary-based approach outlined by Pennebaker et al. (2015) in the German adaptation, DE-LIWC 2015, developed by Meier et al. (2018). This adaptation includes over 80 dictionary categories and encompasses a comprehensive list of 18,711 words, word stems, and various linguistic features tailored to the German language context.

We tested the differences in psycholinguistic features across the levels of reflection coded with qualitative content analysis in the reflective writings, as described in chapter 3.2. Affective processes, negative emotions, and the use of the term “feel” were more prevalent in writings with higher reflective performance (see Table 4). These attributes under affective attributes and perception categories showed a slight but significant impact. The results were *F*_affective processes_ (1, 91) = 4.43, *p* = 0.038, *F*_negative emotion_ (1, 91) = 4.28, *p* = 0.029, and *F*_feel_ (1, 91) = 4.91, *p* = 0.016, respectively. Lastly, we differentiated between the levels of reflection by examining the linguistic features associated with cognitive attributes. Specifically, indicators of cognitive processing, including terms related to discrepancy, certainty, differentiation, negations, and comparisons, were found to be vital in distinguishing between different levels of reflection. The presence and relevance of these cognitive process words were positively correlated with the level of reflection in the writings.

**Table 4:** ANOVA comparing the psycholinguistic attributes of reflective writing at different reflection levels.

Linguistic category	Description	Descriptive reflection	Dialogical reflection	*F*
	*M*/SD	*M*/SD	*M*/SD	
Affective attributes				
Affective processes	5.04/2.32	5.79/2.47	6.21/1.74	4.43^*^
Negative emotion	1.06/0.77	1.30/1.06	1.63/1.17	4.91^*^
Perception				
Feel	0.23/0.41	0.51/0.72	0.62/0.69	6.08^*^
Cognitive attributes				
Cognitive processes	21.05/3.97	23.12/4.21	25.65/3.44	21.92^***^
Discrepancy	1.88/1.18	2.51/1.40	3.38/1.47	19.25^***^
Certainty	3.09/2.27	2.95/1.46	4.16/1.88	4.89^*^
Differentiation	3.69/1.99	4.19/1.72	5.83/2.19	17.93^***^
Negations	0.72/0.92	0.86/0.80	1.61/1.06	13.70^***^
Comparisons	2.08/1.22	2.67/1.03	2.97/1.10	9.71^**^

Statistical significance was reported after subsampling for class imbalance; each group was 31 samples (^***^p < 0.001, ^**^0.001 < *p* < 0.01, ^*^0.01 < *p* < 0.05).

## 4 Discussion

This paper addressed the potential of mixed methods in research on reflective writings in teacher education. This has been explored in three steps: firstly, with a theoretical and empirical overview of research on reflective writing in teacher education; secondly, the description and explanation of qualitative and quantitative research methods (and mixed methods) on reflective writings in teacher education with a specific focus on qualitative content analysis and computer-linguistic methods, and thirdly, the presentation and discussion of the potential of a concurrent mixed method study on reflective writings of pre-service teachers from our lab.

Research on reflective writings represents a theoretically and methodologically multifaceted and interdisciplinary research field. Therefore, the concurrent mixed method approach in our study helped provide a comprehensive analysis of reflective writings. The qualitative content analysis and the computational linguistics methods contributed to analyze reflective writings. Different forms of data were collected simultaneously and then integrated into interpreting the overall results regarding levels of reflection and linguistic features to describe these levels. Furthermore, in this study, we used and analyzed with qualitative content analysis a smaller amount of data (reflective writings of 198 pre-service students) and a more extensive data collection (different linguistic features in those reflective writings) in order to address the different types of research questions. The qualitative content analysis addresses the three levels of reflection while the quantitative analyses are used to test for statistical differences of linguistic features between these three levels of reflection. The integration of findings from the two methods applied provided a more comprehensive insight into the quality of reflective writing. By integrating the methods it was possible to analyze not only the level of reflection in pre-service students’ reflective writings but also how saturated they are and to what extent the level of reflection are related to the linguistic means used in the quantitative analyses. Given these results, suggestions can be derived on which linguistic means increase the likelihood of writing a successful reflection. Based on the linguistic means also assessment of reflective writings may be provided more systematically and economically. Finally, the qualitative and quantitative data may be used for validating purposes. The paper contributes to the broader literature on mixed methods research by offering an illustrative example of how such a combination and integration can occur at the paradigmatic, theoretical, analytical, and interpretative levels. Therefore, as explained in this article and illustrated in our research example, mixed-methods designs have a particular potential for researching the complex field of reflective writing in teacher education. Mixed methods approaches are increasingly used in educational research to obtain the most comprehensive and valid picture of the research subject by combining qualitative and quantitative methods in the sense of complementarity (Hagenauer et al., 2023; Mejeh et al., 2023).

The combination of qualitative and quantitative methods is often justified by the aim to achieve generalization with the quantitative part of the study, while the qualitative part is used to have a deeper insight into single cases or the context of the research subject for a better understanding (Creswell, 2015; Hagenauer et al., 2023). The study presented in this paper illustrates these principles. Specifically, we showed that reflective writing is a complex phenomenon that can only be depicted using several partially intertwined theoretical models (Bengtsson, 1995; Calderhead, 1992; Hatton and Smith, 1995) and, consequently, multiple methods (Whyss, 2013). Qualitative content analysis (Gläser-Zikuda et al., 2020; Mayring, 2022), which is predominantly used in the analysis of reflection, has also been proven to be adequate in our study to determine different levels of reflection. As a limitation, the relatively small number of reflective writings from just one teacher-education course at one single university has to be critically mentioned. The quantitative method applied in our study for analyzing linguistic features (LIWC2015) (Pennebaker et al., 2015) also delivered relevant results, especially in combination with the levels of reflection determined with qualitative content analysis. Therefore, it was possible to identify linguistic attributes that may be assigned to different levels of reflection and to test how the linguistic means differ between the levels of reflection.

One of the main challenges in mixed method research is to draw a common conclusion from the results obtained from the different parts of the mixed methods study at the end of data collection and data analysis – the integration takes place at the level of common interpretation and conclusion. It should be mentioned that the aim is not to report and interpret the results from each part one after the other in the sense of a mere addition, but rather to relate the qualitative and quantitative results and their interpretations to one another in order to conclude from the possibly controversial and conflicting results (Schoonenboom, 2019) to gain a deeper understanding of a particular phenomenon (Greene, 2007). In our study, the levels of reflection determined by using a qualitative content analysis were used in a second step to assign the linguistic attributes to these levels. The combination of the two methods applied in this study was therefore carried out one after the other, i.e., the first step (qualitative analysis) represents a necessary pre-condition for the second step (quantitative linguistic analysis).

As described, reflection is an essential concept for the professional development of teachers (Korthagen, 2001). There is a growing application of approaches to support reflection in teacher education, such as reflective writings, and it is challenging to analyze and adequately assess reflective writing from the viewpoint of teacher educators (Poldner et al., 2014; Ryan, 2013). Up to now, most of the studies have predominantly relied on the one hand on qualitative content analysis and, on the other hand, on quantitative word counts or automated evaluation methods, leaving a gap in covering larger samples or focusing on reflection from a subjective perspective, respectively. Addressing this gap, our study shows, based on a mixed-method approach, that the integration of these different research methods is powerful in analyzing the relationship among different levels of pre-service teachers‘reflection and linguistic features. Finally, by incorporating a mixed-method approach, this study contributes to the future development of evaluation methods to capture, at the same time, analytical depth and multiperspectivity of reflective writing. It becomes clear that research on reflective writing in teacher education – and in the study presented in this paper – gains from applying mixed methods. However, researchers need to understand when and how to use quantitative, qualitative, and mixed methods research designs to fully benefit from their potential (Hagenauer et al., 2023; Teddlie and Tashakkori, 2009).

Next, to methodological considerations, implications for implementing and fostering reflective writing in teacher education can finally been drawn. In line with other studies (Fütterer, 2019; Fütterer et al., 2024; Kember, 1999), the results of our research show that most reflective writings lack quality. Therefore, it is necessary to revisit and improve teacher education programs by incorporating specific training to support pre-service teachers in gaining and deepening reflective skills for a higher quality of reflection concerning their professional development.

## 5 Conclusion

Reflection connects theoretical knowledge with practical experience, which is essential for the professional development of (pre-service) teachers. However, effectively facilitating pre-service teachers’ reflective abilities poses substantial challenges within teacher education, notably the difficulties in precisely analyzing and in a second step evaluating reflective writing. Research on pre-service teachers’ reflective writing has focused on qualitative content analysis or quantitative methods. This study introduces a concurrent mixed method approach to afford a more comprehensive investigation into the levels of reflection, linguistic features of representation, and their interconnections, aiming to bridge the gaps identified in existing research. The findings indicate that pre-service teachers generally exhibited low levels of reflection. However, the cognitive and affective linguistic features in their reflective writing emerged as significant indicators of the reflection level. By employing a mixed method approach, this study not only reveals the substantial methodological benefits but also significantly enriches the body of research on reflective writing in teacher education.

## Data Availability

The original contributions presented in the study are included in the article/supplementary materials, further inquiries can be directed to the corresponding author.

## Appendix

**Table A1:** Example for disagreement and agreement for inter-coder reliability check (translated from German).

**Example 1: Disagreement** Rater 1 (overall rating on level 2) Finally, in contrast to the feedback form, my justification for the diagnostic methods to be used was very brief. While I had included my explanations in a continuous text, the justifications in the feedback were listed as a separate point and differed greatly from my results. (2×1) Overall, it can be said that my answers differed greatly from those on the feedback form. In my opinion, this is possibly due to the fact that I had difficulties with the task in this subject area, which I only really realized when I compared them with the feedback sheet. As I was still able to give my own answers to the questions, I did not use the tips on the assignments, which would have been useful in hindsight. (3×2) In addition, the self-study made it difficult for me to fully grasp the content of this subject area. This also explains why my answers differed from those on the feedback sheet. (2×2)	Rater 2 (overall rating on level 1) Finally, in contrast to the feedback form, my justification for the diagnostic methods to be used was very brief. While I had included my explanations in a continuous text, the justifications in the feedback were listed as a separate point and differed greatly from my results. (2×0) Overall, it can be said that my answers differed greatly from those on the feedback form. In my opinion, this is possibly due to the fact that I had difficulties with the task in this subject area, which I only really realized when I compared them with the feedback sheet. As I was still able to give my own answers to the questions, I did not use the tips on the assignments, which would have been useful in hindsight. (3×2) In addition, the self-study made it difficult for me to fully grasp the content of this subject area. This also explains why my answers differed from those on the feedback sheet. (2×1)
**Example 2: Agreement** Rater 1 (overall rating on level 1) The learning modules were easy to understand and good for learning, there were a few problems with the videos, which is why I only read through the slides and did without the videos. Nevertheless, I got on well with the learning material (2×1) I was able to complete the tasks well. For the first task, I still had to look briefly at my notes as I had worked on the slides a long time ago. (2×1)	Rater 2 (overall rating on level 1) The learning modules were easy to understand and good for learning, there were a few problems with the videos, which is why I only read through the slides and did without the videos. Nevertheless, I got on well with the learning material (2×1) I was able to complete the tasks well. For the first task, I still had to look briefly at my notes as I had worked on the slides a long time ago. (2×1)
